# The validity, reliability, and psychometric properties of the Turkish version of the Digital Amnesia Scale

**DOI:** 10.3389/fpsyg.2026.1775425

**Published:** 2026-04-08

**Authors:** Nevin Günaydın, Mehmet Ali Cengiz, Yaşar Şekerci, Emre Dünder

**Affiliations:** 1 Department of Psychiatric Nursing, Faculty of Health Sciences, Ordu University, Ordu, Türkiye; 2 Department of Mathematics and Statistics, College of Science, Imam Mohammad Ibn Saud Islamic University (IMSIU), Riyadh, Saudi Arabia; 3 Department of Public Relations, Faculty of Communication, Galatasaray University, Istanbul, Türkiye; 4 Department of Statistics, Faculty of Science, Ondokuz Mayıs University, Samsun, Türkiye

**Keywords:** digital addiction, digital amnesia, mental health, public health, reliability, scale adaptation, validity

## Abstract

**Introduction:**

The widespread use of digital technologies has significantly influenced individuals’ cognitive processes and behavioral patterns. Alongside this change, the concept of digital amnesia, defined as the tendency to offload information onto digital devices, has emerged. This study aimed to adapt the Digital Amnesia Scale (DAS) to Turkish and evaluate its validity and reliability in the Turkish cultural context.

**Methods:**

This methodological study was conducted between September 1, 2025, and October 30, 2025 with individuals aged 18 years and older. A total of 205 students who consented to participate formed the study sample. Data were collected using a Personal Information Form, the Digital Addiction Scale, and the Digital Amnesia Scale. The scale’s validity was assessed through content, construct, and criterion-related validity analyses, while reliability was evaluated using Cronbach’s alpha coefficients. Descriptive statistics, reliability analyses, correlation analyses, and confirmatory factor analysis (CFA) were employed for statistical evaluation.

**Results:**

Confirmatory factor analysis indicated that the original three-factor structure of the scale was preserved. Unstandardized factor loadings ranged between 0.365 and 1.371, while standardized factor loadings ranged between 0.211 and 0.845. Cronbach’s alpha for the overall scale was 0.844, with subdimension values ranging from 0.713 to 0.864. Criterion-related validity analyses revealed a moderate, positive, and significant correlation between the Digital Amnesia Scale and the Digital Addiction Scale.

**Conclusion:**

The Turkish version of the Digital Amnesia Scale was found to be a valid and reliable measure in adult populations. The scale can be used in public health–based research to assess digital behaviors and cognitive health indicators.

## 1 Introduction

As digital technologies have become increasingly embedded in everyday life, individuals have begun to rely on external devices for storing and retrieving information — a strategy conceptualized as cognitive offloading (Shanmugasundaram and Tamilarasu, 2023). While this approach may reduce cognitive load and enhance short-term efficiency, it may also weaken the habitual use of internal memory systems over time. This shift has been described as digital amnesia, referring to the reduced ability to retain information due to reliance on digital devices (Beaudoin et al., 2024; Baron, 2021; Geetha et al., 2025). Within the framework of transactive memory theory (Wegner, 1987), delegating memory processes to technological tools may diminish reliance on personal mnemonic systems and increase vulnerability to attentional fragmentation and memory encoding difficulties (Călinescu, 2024; Skulmowski, 2023; Grinschgl et al., 2021). Prior research has linked such changes to impairments in attention, working memory, and long-term memory processes, as well as to structural and functional alterations in brain regions associated with memory and executive functioning, including the prefrontal cortex and hippocampus (Grinschgl et al., 2021; Chao et al., 2020; Das and Menon, 2021; Marciano et al., 2021; Korte, 2020; Manwell et al., 2022; Kanbay et al., 2025). Excessive dependence on digital devices may therefore contribute to reduced use of internal memory resources and to the emergence of digital amnesia as a cognitive consequence of sustained cognitive offloading (Kanbay et al., 2025; Musa and Ishak, 2021; Deckker and Sumanasekara, 2025; Clemente-Suárez et al., 2024; Yousef et al., 2025).

Popularized by the Kaspersky Lab report (2015), the term highlights that overreliance on digital devices may reduce individuals’ perceived responsibility to remember and may contribute to subtle declines in certain cognitive functions (Geetha et al., 2025; Călinescu, 2024; Musa and Ishak, 2021). Increased screen time has also been associated with impatience, attentional difficulties, sleep disturbances, psychological distress, and negative impacts on social relationships (Manwell et al., 2022; Deckker and Sumanasekara, 2025; Ali et al., 2024; Nakshine et al., 2022; Ratan et al., 2021). Among adolescents and young adults’ groups characterized by intensive device use digital amnesia has become a growing concern for educational and health authorities (Manwell et al., 2022; Musa and Ishak, 2021; Tanghian, 2024). Recent findings show that continuously storing information on digital devices may result in less effective long-term memory encoding and greater susceptibility to forgetting (Marciano et al., 2021; Yousef et al., 2025; Oswald et al., 2020).

These global trends are similarly evident in Turkey. Digital device and internet use in the country, particularly among younger populations, exceeds OECD and European averages. Recent national reports indicate extremely high rates of smartphone ownership and internet access, and that digital skills among adolescents and young adults surpass European norms (Evci, 2022; Kursun and Turgut, 2020). Increased dependence on digital knowledge sources, elevated screen time, and heightened distractibility have been shown to negatively affect academic performance, psychological well-being, and social functioning (Kanbay et al., 2025; Evci, 2022; Boz and Dinç, 2023; Bulut and Gokce, 2023; Doruk et al., 2023). Digital media and smartphone overuse have also been linked to poorer sleep quality, attentional problems, psychological symptoms, and social isolation (Bulut and Gokce, 2023; Doruk et al., 2023; Kılıç et al., 2025). According to 2025 data, 92% of the population in Turkey has active mobile internet access, and daily screen time averages nearly 7 h, underscoring the widespread dependence on digital devices (DataReportal, 2025). These usage patterns position Turkey as a population at notable risk for digital amnesia.

Given the high prevalence of digital device use in Turkey, there is an increasing need for culturally appropriate assessment tools to evaluate the cognitive consequences of digital behavior. The Digital Amnesia Scale (DAS) offers a promising framework for assessing cognitive tendencies related to digital amnesia within the Turkish population (Kanbay et al., 2025; Babaoğlu et al., 2025). However, measurement tools used to assess digital amnesia remain limited, highlighting a clear gap in the literature (Babaoğlu et al., 2025; Robert and Kadhiravan, 2022). In recent years, a growing body of empirical research has examined digital amnesia and related cognitive consequences across diverse populations (Shanmugasundaram and Tamilarasu, 2023; Beaudoin et al., 2024; Kanbay et al., 2025; Musa and Ishak, 2021; Tanghian, 2024; Babaoğlu et al., 2025; Robert and Kadhiravan, 2022). These contemporary findings underscore the increasing relevance of the construct and highlight the importance of further psychometric validation studies in different cultural contexts. Therefore, validating and assessing the reliability of the Turkish version of the Digital Amnesia Scale represents a critical step toward evaluating the cognitive implications of digital technology use in the contemporary digital age.

## 2 Methods

This methodological study was conducted between September 1, 2025, and October 30, 2025, aimed at examining the Turkish validity and reliability of the Digital Amnesia Scale (DAS) in adults.

### 2.1 Study design and sample

The study was conducted in a province in northern Turkey between September and October 2025 among individuals aged 18 years and older. Inclusion criteria were being 18 years or older, using at least one digital device, and having no condition that would prevent completion of the survey. Exclusion criteria included being under 18 years of age, not using a digital device, or having a health condition that would hinder completion of the survey.

For the cultural adaptation of scales and in validity and reliability studies, sample size adequacy is typically ensured by having the number of participants at least 5–10 times the number of items in the scale (sample size/items in the scale: 20*10 = 200) (Aguinis and Harden, 2010; Hair Jr, 2006; Tabachnick and Fidell, 2013). Accordingly, 205 participants selected through convenience sampling were included in the study.

For scale adaptation studies, sample size adequacy is commonly evaluated based on both item-to-participant ratios and recommended sample size classifications. A frequently used guideline suggests including at least 5–10 participants per item (Aguinis and Harden, 2010; Hair Jr, 2006; Tabachnick and Fidell, 2013). In addition, commonly cited criteria indicate that a sample size of around 200 is generally considered acceptable for scale validation studies (Comrey and Lee, 1992; Şencan, 2005). Based on these recommendations, a minimum sample of 200 participants was targeted for the 20-item scale, and 205 individuals were included in the study.

Using G*Power 3 (Faul et al., 2007), a power analysis was conducted based on the correlation analysis for criterion validity, assuming a medium effect size (*r* = 0.30) as suggested by Cohen (1988). At a significance level of *α* = 0.05, a sample of *n* = 205 participants achieved a power of (1–*β*) = 0.993. Consequently, the study sample consisted of 205 adults aged 18 years and above recruited through convenience sampling.

### 2.2 Data collection

Data were collected using the Personal Information Form, the Digital Amnesia Scale (DAS), and the Digital Addiction Scale. The first section of the survey included sociodemographic characteristics such as age, gender, income level, department, and class level, while the second section comprised the relevant scales.

#### 2.2.1 Personal Information Form

This form consisted of 15 items addressing participants’ sociodemographic characteristics (e.g., age, gender, class, family income, parents’ educational level, age at which they started using a mobile phone).

#### 2.2.2 Digital Addiction Scale

Developed by Kesici and Tunç (2018), the scale consists of 19 items across five factors: excessive use (5 items), relapse (3 items), disruption of life flow (4 items), mood (4 items), and inability to quit (3 items). Each item is rated on a 5-point Likert scale, ranging from 1 to 5, with total scores ranging from 19 to 95. Higher scores indicate higher levels of digital addiction. The Cronbach’s alpha coefficient of the scale was reported as 0.87 (Kesici and Tunç, 2018), and in the present study, it was found to be 0.934, indicating excellent internal consistency.

#### 2.2.3 Digital Amnesia Scale (DAS)

Developed by James Roberts (Robert et al., 2024), the scale consists of 20 items measuring three dimensions of digital amnesia: digital distraction (digdist), digital addiction (digadd), and digital detox (digdet). Digital distraction comprises nine items, digital addiction six items, and digital detox five items, all of which are reverse-scored. Items are rated on a 5-point Likert scale, ranging from 1 (Never) to 5 (Always) (e.g., “I rely on my phone to remember my parents’ phone numbers”). Higher scores indicate a greater likelihood of developing digital amnesia. The scale’s Cronbach’s alpha was reported as 0.70.

**Figure 1:**
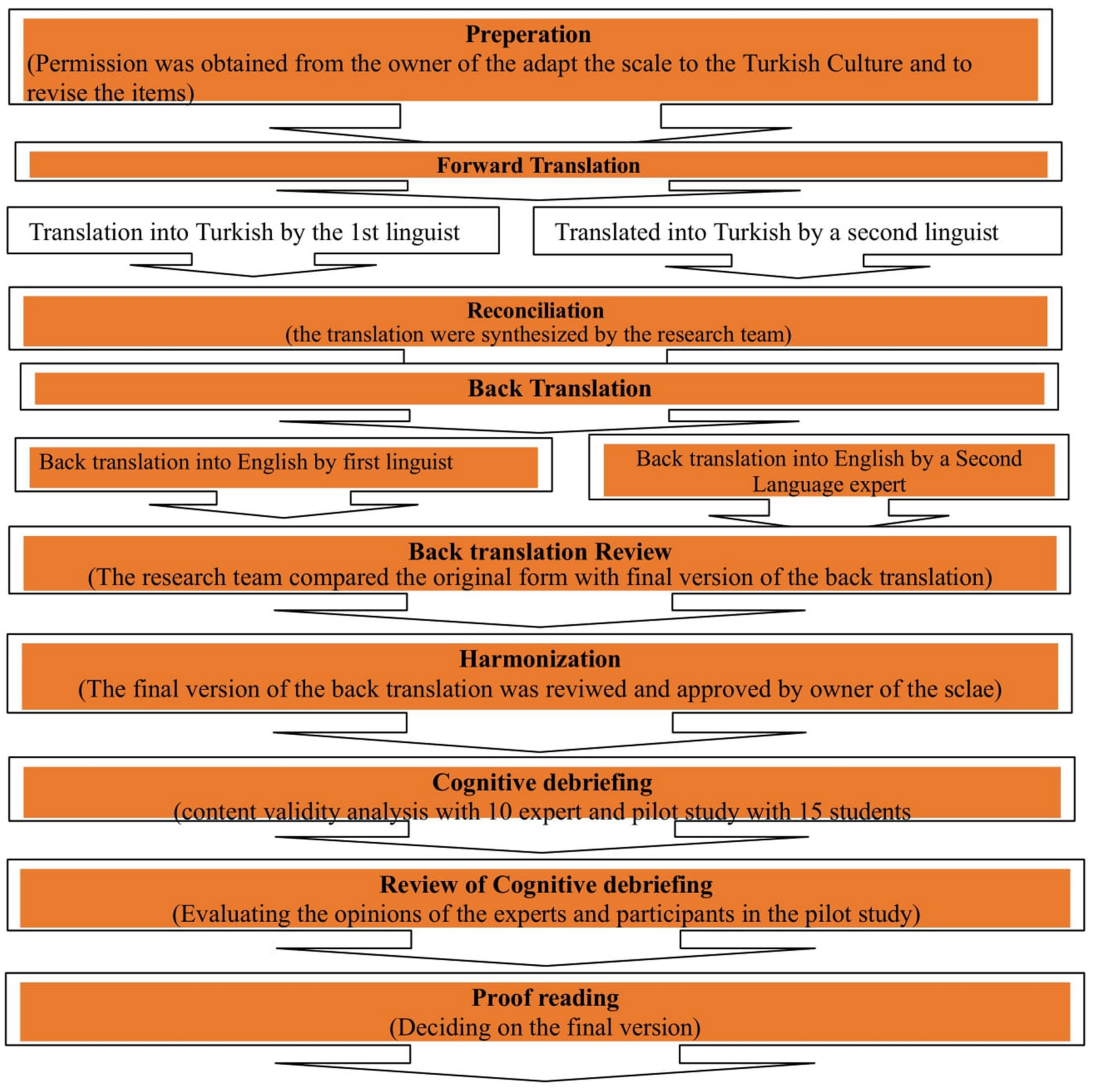
According to ISIPOR, the process of adapting the Digital Amnesia Scale (DAS) adult scale to Turkish culture.

The Turkish adaptation and validation process of the scale was structured according to the ISPOR (International Society for Pharmacoeconomics and Outcomes Research) guidelines (Figure 1; Wild et al., 2006). Initially, two Turkish-language experts from the Department of Basic English at the School of Foreign Languages of a state university translated the original items into Turkish. Subsequently, nine academics specializing in internet addiction studies reviewed and evaluated the Turkish version at the item level. Based on expert feedback on the items’ equivalence to the original version and their appropriateness for the target population, the original scale was deemed suitable. The items were subsequently revised in accordance with expert recommendations. To assess clarity, each item was rated on a 5-point Likert scale: 1 = “Strongly Disagree,” 2 = “Disagree,” 3 = “Neutral,” 4 = “Agree,” and 5 = “Strongly Agree.”

To ensure accuracy and linguistic coverage, three professors backtranslated the Turkish version into English. Additionally, a faculty member in Turkish Language and Literature evaluated the final Turkish version. After completing these checks, a pilot test was conducted with 20 students to assess the readability of the Turkish scale. Data obtained from the pilot group were not included in the main Turkish validity and reliability study (Figure 1).

### 2.3 Process

#### 2.3.1 Adaptation of the DAS into Turkish and content validity

##### 2.3.1.1 Linguistic validity

In this study, the adaptation of the DAS scale into Turkish, as well as its validity and reliability assessment, was conducted in accordance with the ISPOR guidelines (Wild et al., 2006). Two Turkish-language experts employed in the School of Foreign Languages, Basic English Department, of a public university initially translated the scale items into Turkish. The Turkish version was then back-translated into English by a bilingual linguist in both languages and cultures. After consulting with the original scale developer and making the necessary revisions, the final Turkish version of the scale items was reviewed and finalized by a lecturer in Turkish language and literature.

#### 2.3.2 Content validity

After confirming the linguistic validity of the DAS scale, multiple experts were consulted to assess its content validity (Büyüköztürk, 2014). Following their recommendations, the Content Validity Index (CVI) of all scale items in the Turkish version was evaluated using the Davis technique by 9 academics who work with university students (Erkal, 2014). The experts were asked to assess the appropriateness of the scale items for translation from their original forms and for the target population. Based on the feedback provided by the experts, the items were revised accordingly. Each item’s comprehensibility was rated on a 5-point Likert scale: 1 = strongly disagree, 2 = disagree, 3 = neither agree nor disagree, 4 = agree, and 5 = strongly agree. Subsequently, the Turkish form was translated into English by three professors to ensure accuracy and linguistic coverage for the next phase of the study.

#### 2.3.3 Pilot study

After completing all preliminary checks, the final version of the scale was administered to a group of 20 individuals who were similar in characteristics to the main study sample but were not part of the primary research. These participants were asked to evaluate the items in terms of clarity, relevance, and readability. Based on their feedback, the final version of the scale was revised as necessary.

#### 2.3.4 Criterion validity

To evaluate criterion validity, the Digital Addiction Scale was administered alongside the Digital Amnesia Scale, and the relationships between the scores of the two scales were examined using correlation analysis.

Construct validity is used to determine the theoretical and practical alignment of a scale (Aktürk et al., 2016; Akgül, 2003). The strength of the correlation was interpreted as follows: 0–0.49, low; 0.50–0.749, moderate; and 0.75–1.00, high.

### 2.4 Ethical approval

The study was approved by the Clinical Research Ethics Committee of Galatasaray University (Approval No.: 2025/014-E-65364513-650-97894, dated 08/05/2025). Institutional permission for the study was also obtained (Permission No.: E-37748962-010.99-1184081/02.09.2025). All procedures performed in this study involving human participants were conducted in accordance with the ethical standards of the institutional research committee and with the 1964 Helsinki Declaration and its later amendments.

Written informed consent was obtained from all participants prior to data collection.

Prior to the translation of the scale into Turkish, James Roberts (Robert et al., 2024), the developer and corresponding author of the scale, provided written consent via email. The purpose of the study was explained to participants, and it was emphasized that participation was entirely voluntary, after which their written consent was obtained. The completed forms were carefully reviewed, and those that were incomplete or incorrectly filled were excluded. Ultimately, 205 forms were used to evaluate the validity and reliability of the instrument.

### 2.5 Data analysis

This section presents the statistical evaluations related to the Turkish adaptation of the Digital Amnesia Scale. Descriptive statistics, reliability analyses, correlation analyses, and confirmatory factor analysis (CFA) were performed. To assess the internal consistency of the Turkish version of the Digital Amnesia Scale, Cronbach’s alpha and Omega coefficients were calculated. Construct validity of the Turkish-adapted scale was examined using CFA with the Diagonally Weighted Least Squares (DWLS) method (Nestler, 2013; DeJohn et al., 2025).

**Figure 2:**
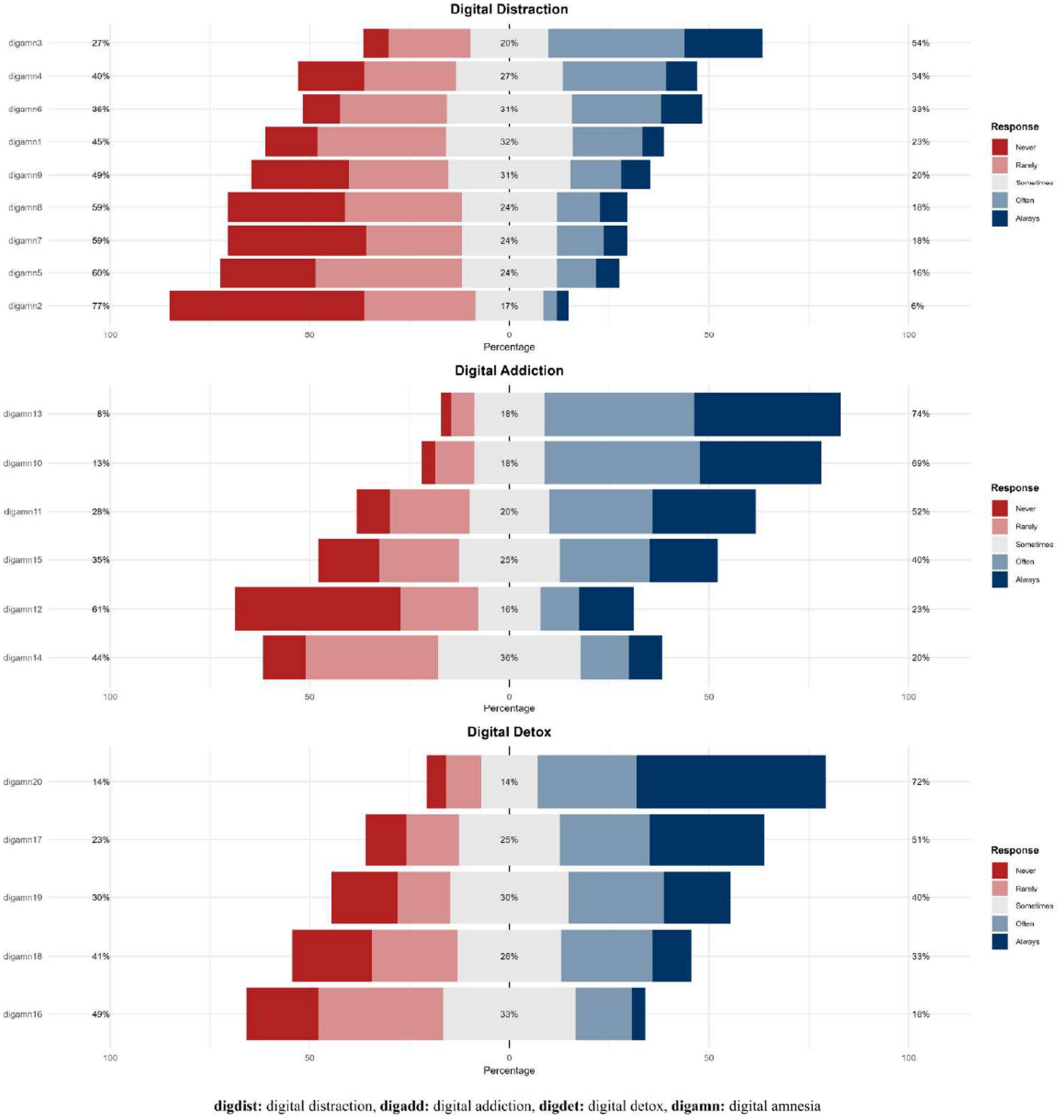
Distribution of responses according to Digital Amnesia dimensions.

Confirmatory factor analysis was evaluated using standardized factor loadings and model fit indices (*χ*^2^/df, CFI, TLI, GFI, AGFI, NFI, IFI, RMSEA) in line with established SEM reporting guidelines. Standardized estimates were used for model interpretation and are presented in Figure 2, whereas unstandardized estimates are reported separately in Table 1. For criterion validity, the Digital Addiction Scale was used as a parallel measure, and its association with the study scale was examined using Pearson correlation coefficients. The normality of score distributions was assessed using skewness (±2) and kurtosis (±2) indices (Demir, 2022; Zadworna and Stetkiewicz-Lewandowicz, 2023). As the normality assumption was satisfied, parametric techniques were applied in correlation analyses. All statistical analyses were conducted using R software (R Core Team, 2025) with the packages corrplot (Wei and Simko, 2024), ggplot2 (Wickham, 2016), lavaan (Rosseel, 2012), lavaanPlot (Lishinski, 2021), and psych (Revelle, 2023). A significance level of 0.05 was applied in all analyses.

**Table 1:** Statistical results for path coefficients of the Digital Amnesia Scale.

Item	Estimate	Standardized estimate	*z*	*p*
Digital distraction				
digamn1	1	0.779		
digamn2	0.432	0.337	10.455	<0.001
digamn3	0.994	0.775	22.305	<0.001
digamn4	0.906	0.706	21.686	<0.001
digamn5	0.999	0.778	22.071	<0.001
digamn6	0.88	0.686	21.168	<0.001
digamn7	0.760	0.592	18.895	<0.001
digamn8	1.058	0.824	22.801	<0.001
digamn9	0.792	0.617	19.141	<0.001
Digital addiction				
digamn10	1	0.579		
digamn11	1.371	0.794	15.229	<0.001
digamn12	0.365	0.211	5.882	<0.001
digamn13	1.156	0.670	13.97	<0.001
digamn14	0.933	0.540	12.764	<0.001
digamn15	1.350	0.782	15.233	<0.001
Digital detox				
digamn16	1	0.636		
digamn17	0.946	0.602	9.468	<0.001
digamn18	0.818	0.521	9.198	<0.001
digamn19	1.329	0.845	9.556	<0.001
digamn20	0.844	0.537	8.611	<0.001

*z*, z-statistic; *p*, significance. digdist, digital distraction; digadd, digital addiction; digdet, digital detox; digamn, digital amnesia.

## 3 Results

### 3.1 Descriptive findings

A total of 205 participants were included in the study. Although the study included individuals aged 18 years and older, the sample predominantly consisted of university students from a single province in northern Turkey. Therefore, the sample represents primarily young adults rather than the broader Turkish adult population. Regarding gender distribution, 55.6% (*n* = 114) were female, and 44.4% (*n* = 91) were male. In terms of marital status, 59.5% (*n* = 122) of the participants were single, and 40.5% (*n* = 83) were married.

When examining technology usage habits, 34.1% (*n* = 70) of participants reported needing navigation applications on their phones to find addresses. Additionally, 82.4% (*n* = 169) stated that they could remember the phone numbers of their first-degree relatives. Regarding social media usage, 27.8% (*n* = 57) of participants reported using social media for 0–2 h per day, 33.7% (*n* = 69) for 2–4 h, 23.4% (*n* = 48) for 4–6 h, 9.8% (*n* = 20) for 6–8 h, and 5.4% (*n* = 11) for 8 h or more per day. The mean total score of the Digital Amnesia Scale was 58.66 ± 11.94.

**Figure 3:**
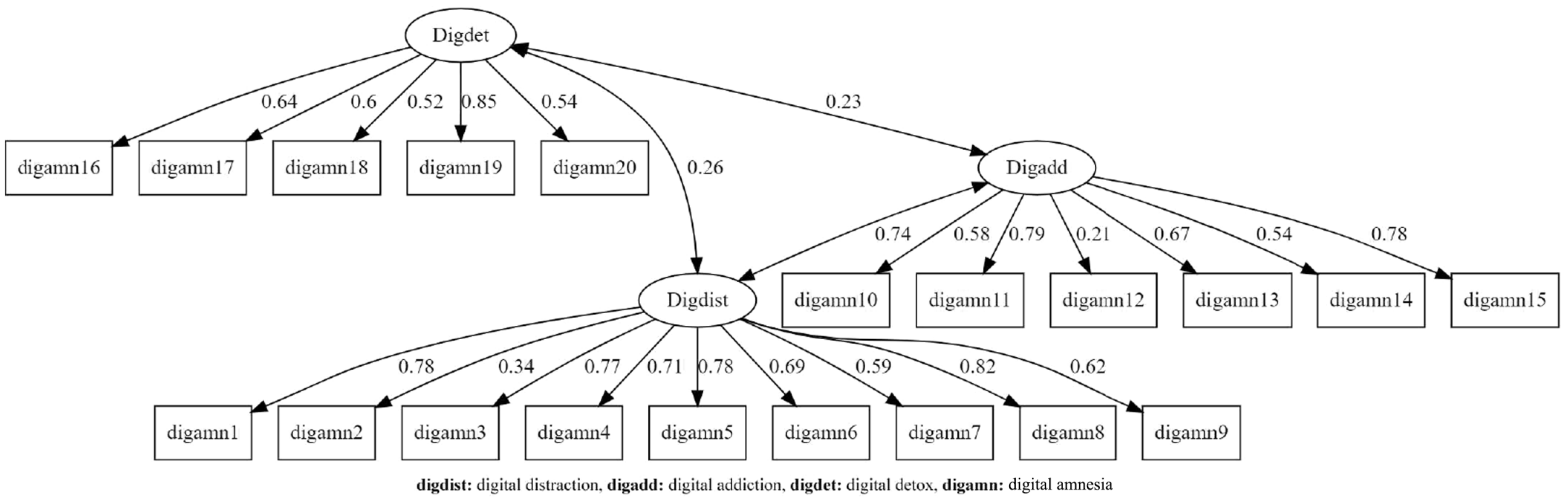
Confirmatory factor analysis (CFA) diagram for the Turkish version of the Digital Amnesia Scale.

Figure 3 presents the item-level response distributions for the subdimensions of the Digital Amnesia Scale. In the figure, the percentages correspond to the combined responses of “never” and “rarely” versus “often” and “always,” while the percentages shown in the center of the graphs represent the “sometimes” responses (Figure 3).

Within the Digital Attention Disruption subdimension, the highest response rate was observed for item digamn3 (“I use my technological devices while in bed”) at 54%, whereas the lowest rate was for item digamn2 (“I use my phone while driving”) at 6%. In the Digital Addiction subdimension, the highest rate was reported for item digamn13 (“I use my phone when I need information”) at 74%, and the lowest for item digamn14 (“It is difficult for me to remember information without the help of technological devices”) at 20%. For the Digital Detox subdimension, among the reverse-scored items, the highest rate was observed for digamn20 (“I usually turn off my phone before going to bed”) at 72%, and the lowest for digamn16 (“I put aside my technological devices while spending time with my family”) at 18%.

Overall, these findings indicate that participants exhibit relatively high behaviors related to digital addiction and attention disruption, whereas digital detox behaviors are comparatively low.

### 3.2 Internal consistency

Reliability analyses were conducted to examine the internal consistency of the Turkish version of the Digital Amnesia Scale. Both item-level reliability indices and overall internal consistency coefficients were evaluated (Table 2).

**Table 2:** Reliability analysis results of the Turkish version of the Digital Amnesia Scale.

Factor/item	CITC	CAIID	Alpha	Omega
Digital distraction				
digamn1	0.752	0.843	0.864	0.867
digamn2	0.457	0.871		
digamn3	0.703	0.849		
digamn4	0.728	0.846		
digamn5	0.787	0.838		
digamn6	0.669	0.852		
digamn7	0.685	0.851		
digamn8	0.798	0.837		
digamn9	0.637	0.857		
Digital addiction				
digamn10	0.613	0.695	0.726	0.749
digamn11	0.720	0.662		
digamn12	0.525	0.762		
digamn13	0.690	0.668		
digamn14	0.671	0.675		
digamn15	0.729	0.658		
Digital detox				
digamn16	0.675	0.659	0.713	0.718
digamn17	0.720	0.651		
digamn18	0.661	0.682		
digamn19	0.747	0.632		
digamn20	0.612	0.699		
Overall results			0.844	0.886

CITC, corrected item-total correlation; CAIID, Cronbach’s alpha if item deleted; alpha, Cronbach’s alpha; omega, McDonald’s omega; digdist, digital distraction; digadd, digital addiction; digdet, digital detox; digamn, digital amnesia.

The corrected item-total correlations exceeded 0.30 across all three subdimensions, indicating a high level of coherence among the scale items. No significant increase in Cronbach’s alpha values was observed when any item was removed from the Digital Distraction (digdist), Digital Addiction (digadd), or Detox (digdet) subdimensions. Consequently, it was concluded that no item in the Turkish version adversely affected internal consistency. The overall internal consistency coefficients of the scale were calculated as Cronbach’s *α* = 0.844 and McDonald’s *Ω* = 0.886, with reliability coefficients for all three subdimensions exceeding 0.70.

These results suggest that the Turkish version of the Digital Amnesia Scale demonstrates high internal consistency and can be considered a reliable measurement tool. For the parallel measure, the Digital Addiction Scale, Cronbach’s *α* = 0.934 and *Ω* = 0.944 were obtained, indicating similarly high internal consistency.

### 3.3 Construct validity

Factor names were standardized across the manuscript to ensure consistency between the text, tables, and figures. Confirmatory Factor Analysis (CFA) was conducted to assess the construct validity of the Turkish version of the Digital Amnesia Scale. The resulting path coefficients are presented in Table 1, and the model fit indices are presented in Table 3. All standardized coefficients are displayed in Figure 2 represent standardized estimates to facilitate interpretation of the factor structure. “All factor loadings shown in Figure 2 are standardized estimates.

In the three-factor structure of the scale, standardized factor loadings ranged from 0.21 to 0.85. All loadings were positive and statistically significant (*p* < 0.001). Except for one item with a relatively lower loading (0.21), factor loadings were at or above the commonly accepted threshold of 0.30, indicating adequate item–factor relationships. These findings suggest that the three-factor structure of the original scale was largely preserved in the Turkish version and that construct validity is supported. Evaluation of the model fit indices (Table 3) indicated good fit, with *χ*^2^/df = 2.264 (Brown, 2015; Ye et al., 2025). Incremental fit indices were also high (GFI = 0.967, CFI = 0.966, AGFI = 0.946, TLI = 0.961, IFI = 0.966, NFI = 0.940), and RMSEA = 0.079 indicated an acceptable level of model fit (Hu and Bentler, 1999; Sun and Xiao, 2023; Kline, 2023). Although model fit indices indicated acceptable to good fit, RMSEA values approaching the upper acceptable boundary suggest that replication studies with larger and more diverse samples are warranted.

**Table 3:** Fit indices calculated for the Turkish version of the Digital Amnesia Scale.

CHISQ	df	CHISQ/df	GFI	CFI	AGFI	TLI	IFI	NFI	RMSEA
378.007	167	2.264	0.967	0.966	0.946	0.961	0.966	0.940	0.079

df, degrees of freedom; GFI, Goodness of Fit Index; CFI, Comparative Fit Index; AGFI, Adjusted Goodness of Fit Index; TLI, Tucker–Lewis Index; IFI, Incremental Fit Index; NFI, Normed Fit Index; RMSEA, root mean square error of approximation. digdist, digital distraction; digadd, digital addiction; digdet, digital detox; digamn, digital amnesia.

These findings demonstrate that the Turkish adaptation of the Digital Amnesia Scale exhibits a well-fitting three-factor structure, confirming its construct validity.

### 3.4 Criterion validity

**Figure 4:**
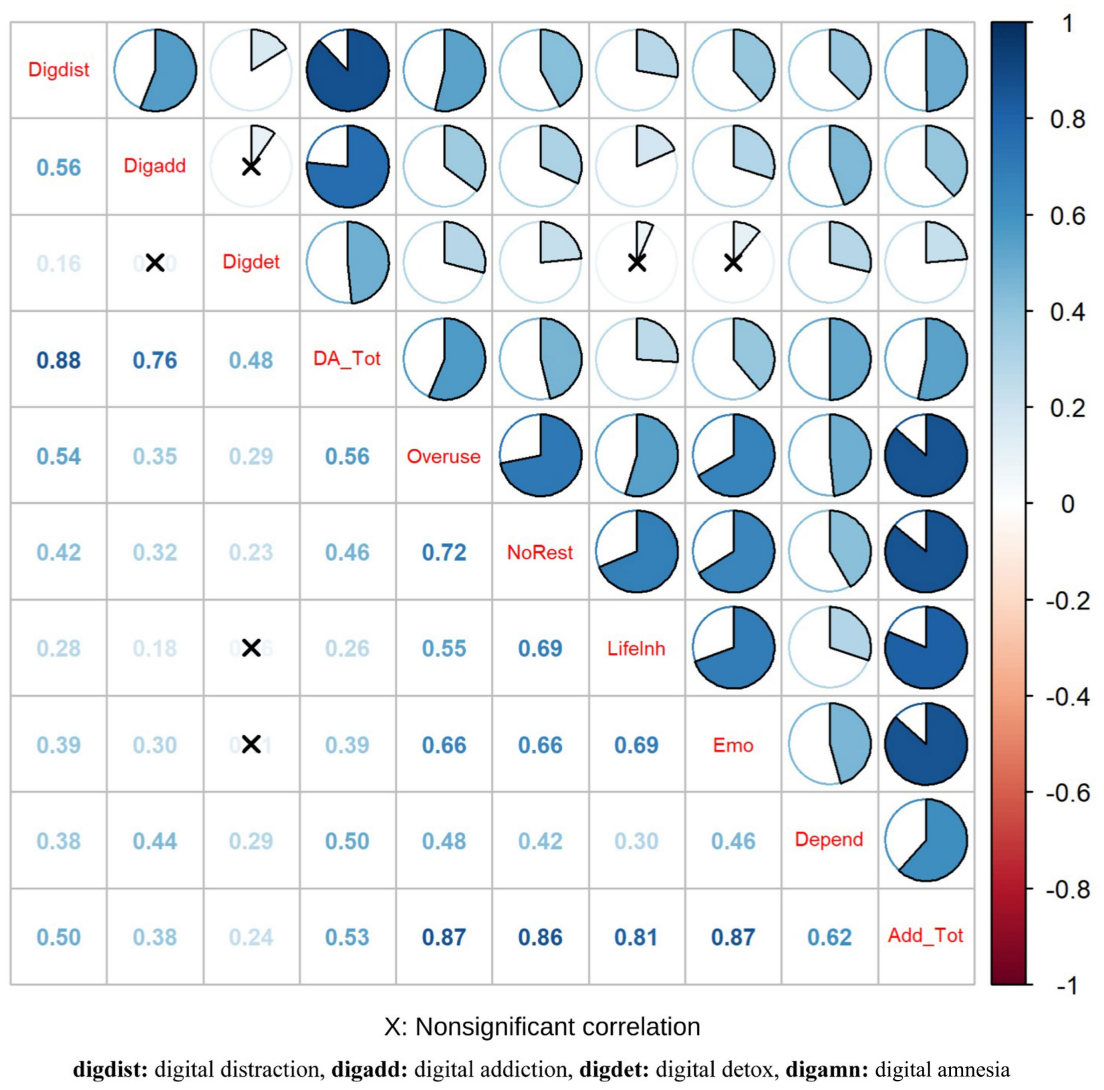
Correlation graphs.

To evaluate criterion validity, the Digital Addiction Scale was administered alongside the Digital Amnesia Scale, and the relationships between the scores of the two scales were examined using correlation analysis (Figure 4).

The results indicated significant positive correlations between the two scales at both the total score and subdimension levels. These findings support the criterion validity of the Turkish version of the Digital Amnesia Scale. All reported statistical values were cross-checked across text, tables, and figures to ensure consistency.

## 4 Discussion

The present findings should be interpreted within the broader literature on cognitive offloading. While digital technologies can alleviate cognitive load and enhance short-term performance, prior research has associated sustained reliance on external devices with alterations in memory processes and tendencies related to digital amnesia (Shanmugasundaram and Tamilarasu, 2023). Thus, recent syntheses also highlight that our findings are part of a broader pattern documented in contemporary research. A comprehensive review on technology-based cognitive offloading and learning shows that externalizing information to digital media often leads individuals to retain mainly the “gist” or the location of information, while deeper long-term encoding is reduced, which is conceptualized as a form of digital externalization and digital amnesia (Skulmowski, 2023). These conclusions are consistent with the behavioral patterns observed in the present dataset, suggesting that everyday externalization of information to smartphones is associated with weaker resources on internal memory resource.

This study examined the psychometric properties of the Turkish version of the Digital Amnesia Scale (DAS) and demonstrated that its three-factor structure is valid and reliable among a Turkish adult sample. Confirmatory factor analysis results (*χ*^2^/df = 2.264, CFI = 0.966, GFI = 0.967, RMSEA = 0.079) are largely consistent with the model fit reported for the original scale (Robert et al., 2024; Brown, 2015; Hu and Bentler, 1999). The significance of all factor loadings indicates that construct validity was preserved during the cultural adaptation process, and the theoretical model of the scale was successfully replicated in the Turkish sample (Kline, 2023). Recent systematic reviews and meta-analyses further support the relationship between intensive digital device use, cognitive offloading, and memory-related difficulties. Contemporary evidence suggests that prolonged reliance on digital technologies is associated with reduced internal memory engagement, attentional fragmentation, and increased vulnerability to digital amnesia–related behaviors (Beaudoin et al., 2024; Deckker and Sumanasekara, 2025; Ali et al., 2024). Neurocognitive studies conducted in recent years indicate that excessive screen exposure may influence functional connectivity between the prefrontal cortex and hippocampus, thereby affecting memory encoding and retrieval processes (Das and Menon, 2021; Yousef et al., 2025). These findings reinforce the conceptual framework underlying the Digital Amnesia Scale and highlight the relevance of assessing digital amnesia as a distinct cognitive phenomenon in contemporary populations.

These findings align with the cognitive offloading literature, which suggests that increasing reliance on digital devices may improve task performance in the short term but weaken internal memory processes over time (Shanmugasundaram and Tamilarasu, 2023; Grinschgl et al., 2021). Growing dependence on external tools can reduce individuals’ confidence in their own memory capacity, thereby perpetuating a cycle that contributes to digital amnesia. Recent systematic reviews and meta-analyses further support this mechanism by showing that intensive and continuous digital engagement is associated with attentional difficulties, cognitive fatigue, and disruptions in self-regulation, while intentional reduction of use (digital/social media detox) can partially restore psychological functioning and cognitive resources (Ramadhan et al., 2024; Schäfer et al., 2024; Mahsan et al., 2023). In particular, a 2024 meta-analysis on digital detox interventions found that structured breaks from social media significantly reduced depressive symptoms and was interpreted as allowing recovery of depleted cognitive and emotional resources (Ramadhan et al., 2024). These syntheses indicate that patterns of high addiction and low disengagement have been discussed in the literature as part of broader cognitive and behavioral profiles linked to impaired attentional control and reduced reliance on internal resources (Ramadhan et al., 2024; Schäfer et al., 2024; Wols et al., 2024). A similar pattern was descriptively observed in our sample, which showed high scores for digital attention disruption and digital addiction, alongside low scores for digital detox behaviors.

The overall Cronbach’s alpha (*α* = 0.844) and McDonald’s Omega (*Ω* = 0.886) values indicate high internal consistency, exceeding the reliability coefficients reported for the original scale (α = 0.70). Subdimension reliability coefficients above 0.70, including the reverse-scored Digital Detox items, further support the Turkish version of the scale as a reliable instrument for both research and clinical applications. Corrected item-total correlations were all above 0.30, and no increase in reliability was observed when any item was removed, indicating that the item structure functions cohesively and in a balanced manner.

Descriptive findings indicated relatively higher scores in digital attention disruption and digital addiction dimensions, and comparatively lower scores in digital detox behaviors within this sample. These results reflect observed behavioral tendencies in the present cross-sectional dataset and should not be interpreted as evidence of causal or developmental effects. High endorsement rates for items such as “I use my phone when I need information” (digamn13) and “I use my devices before going to bed” (digamn3) are consistent with the intensive access to digital technologies and the integration of smartphone use into daily life among young adults in Turkey (Kursun and Turgut, 2020; Bulut and Gokce, 2023). Conversely, the low endorsement of putting devices aside while spending time with family (digamn16) may reflect the normalization of continuous technology use within this sample even in social contexts. These findings suggest an alignment between reported digital behavior patterns and DAS scores within the sample; however, causal interpretations cannot be made due to the cross-sectional design.

Positive, moderate correlations between the Digital Amnesia Scale and the Digital Addiction Scale indicate that digital amnesia represents a conceptual domain related to, yet distinct from, digital addiction. This finding aligns with previous research suggesting that continuous access to digital technologies increases reliance on external resources in cognitive processes (Shanmugasundaram and Tamilarasu, 2023; Greenwood and Quinn, 2017). Theoretical frameworks on cognitive offloading propose that the progressive transfer of responsibility for information storage and retrieval to digital tools may reduce internal memory processes over time (Beaudoin et al., 2024; Grinschgl et al., 2021). Although the observed moderate correlations provide support for criterion-related validity, it is important to interpret these findings with caution. The Digital Addiction Scale represents a conceptually adjacent construct, and some degree of theoretical overlap between digital addiction and digital amnesia may be expected, particularly regarding excessive reliance on digital devices. However, digital amnesia specifically refers to cognitive offloading tendencies and potential weakening of internal memory processes, which are not fully captured by measures of behavioral addiction alone. In addition, the present study did not include objective cognitive performance measures (e.g., memory or attention-based tasks) as external validation criteria. Therefore, conclusions regarding the cognitive specificity of the construct should be considered preliminary. Future studies incorporating neurocognitive assessments or behavioral memory measures would further strengthen the criterion validity evidence of the scale.

The low levels of digital detox behaviors observed in the Turkish sample suggest that this self-reinforcing cycle is strengthened in the local context, supporting the effectiveness of the DAS as a tool for assessing such tendencies. Globally, approximately one in four individuals is at risk of digital addiction, with higher prevalence rates reported in middle- or low-income countries and in Eastern Mediterranean regions, including Turkey (Meng et al., 2022). These results provide evidence for the criterion validity of the scale and highlight the importance of monitoring digital amnesia, measuring its levels, and developing integrated intervention strategies addressing both digital addiction and digital amnesia.

These findings also make a significant contribution in the context of Turkey. Young adults in Turkey are known to have high levels of technology ownership, long screen times, and intensive digital media use. Consequently, the systematic assessment of levels of digital amnesia provides a foundation for new research models that examine how cognitive processes are influenced by digital behaviors, particularly in fields such as education, psychology, and health. The preservation of the scale’s three-factor structure in the Turkish context suggests that the components of digital attention disruption, addiction, and detox operate through similar cognitive mechanisms across cultures. Recent national and international reports indicate that Turkey remains among the countries with the highest levels of smartphone penetration and daily screen time, particularly among young adults (DataReportal, 2025). Studies published within the last 5 years have shown that excessive digital media use in this population is associated with decreased attention span, impaired academic functioning, and increased psychological distress (Bulut and Gokce, 2023; Doruk et al., 2023). In this context, the availability of a valid and reliable Turkish version of the Digital Amnesia Scale provides an important tool for monitoring emerging cognitive risks related to digital technology use.

However, this study has some limitations. The sample comprised only a specific group, which may limit the generalizability of the findings and highlights the need to examine digital amnesia levels across broader age ranges and diverse socioeconomic backgrounds. In addition, the cross-sectional design does not allow for causal inferences; longitudinal studies would provide stronger evidence for understanding changes in digital behaviors over time.

Overall, this study demonstrates that the Turkish version of the DAS is a valid and reliable instrument and provides a solid psychometric foundation for future research on digital amnesia in Turkey. The scale may be used in research, public health, and preventive mental health contexts to assess digital amnesia–related cognitive tendencies and to evaluate intervention programs promoting healthy digital technology use. Nevertheless, given the cross-sectional design and relatively homogeneous sample, broader cognitive interpretations should be made cautiously and require further validation in diverse populations.

### 4.1 Limitations

This study has several limitations. The sample consisted predominantly of young adults recruited from a single geographical region, which may limit the representativeness of the findings for the broader Turkish adult population. Future studies should include nationally representative and more heterogeneous samples. Its cross-sectional design restricts generalizability and precludes causal inferences. Therefore, future research is recommended to re-evaluate the scale across different demographic and socioeconomic groups in Turkey and to employ longitudinal designs to examine temporal dynamics in digital amnesia.

In addition, although the present study provided evidence regarding internal consistency, construct validity, and criterion-related validity, other important psychometric properties were not examined. Specifically, test–retest reliability was not assessed; therefore, the temporal stability of the Turkish version of the scale remains to be established. Furthermore, measurement invariance across demographic groups (e.g., gender) was not tested, and convergent and discriminant validity were not evaluated using theoretically distinct non-addiction constructs (e.g., memory performance, attention, or executive functioning measures). Accordingly, the findings should be interpreted as providing initial evidence of validity rather than a comprehensive psychometric evaluation of the scale.

### 4.2 Suggestions for future research

Future studies should evaluate the test–retest reliability and longitudinal stability of the Turkish version of the Digital Amnesia Scale in order to better investigate its psychometric qualities. Research with a wider range of age groups, professional backgrounds, and clinical samples would improve our comprehension of the scale’s generalizability. Furthermore, measurement invariance across age, gender, socioeconomic status, and degrees of digital technology use may be investigated in future studies.

Beyond psychometric evaluation, future studies should investigate the underlying cognitive and neurocognitive mechanisms associated with digital amnesia. Experimental designs manipulating digital offloading behaviors (e.g., allowing versus restricting device access during information encoding) may help clarify causal relationships between external memory reliance and internal memory performance. Incorporating objective cognitive performance measures, such as working memory tasks, attention paradigms, or executive functioning assessments, would strengthen the construct validity of digital amnesia and provide more direct evidence regarding its cognitive specificity.

Neurocognitive research integrating neuroimaging or electrophysiological methods may further elucidate the neural correlates of digital amnesia, particularly regarding functional connectivity between prefrontal and hippocampal regions. Cross-cultural comparative studies would also be valuable in determining whether digital amnesia manifests similarly across societies with varying levels of technological penetration and cultural norms related to technology use.

Finally, longitudinal and intervention-based research should examine whether structured digital detox programs or cognitive training interventions can modify digital amnesia–related tendencies over time. Such studies would contribute not only to theoretical understanding but also to the development of evidence-based prevention and intervention strategies promoting sustainable digital well-being.

## 5 Conclusion

This study provides initial psychometric evidence supporting the validity and reliability of the Turkish version of the Digital Amnesia Scale in a sample of young adults. The findings indicate that the scale demonstrates a clear factor structure and satisfactory internal consistency, supporting its use as a psychometrically sound measurement tool. The results are consistent with previous research on cognitive offloading and digital technology use, contributing further empirical support to the growing literature on intensive digital engagement.

The Turkish version of the Digital Amnesia Scale has potential applications in educational, clinical, public health, and research contexts. In educational settings, it may assist in identifying individuals who exhibit high levels of cognitive offloading and digital dependence. In clinical and counseling contexts, it may support the assessment of cognitive patterns associated with excessive technology use. In research settings, the scale may facilitate investigations into the cognitive and psychological mechanisms underlying digital behavior and may be used to evaluate intervention outcomes aimed at promoting digital well-being.

Future research should examine the test–retest reliability of the scale and evaluate its applicability across different age groups, regions, and cultural contexts to further strengthen its generalizability, temporal stability, and cross-cultural robustness.

## Data Availability

The raw data supporting the conclusions of this article will be made available by the authors, without undue reservation.
