# Correction: The validity, reliability, and psychometric properties of the Turkish version of the Digital Amnesia Scale

**DOI:** 10.3389/fpsyg.2026.1865916

**Published:** 2026-05-29

**Authors:** Nevin Günaydin, Mehmet Ali Cengiz, Yaşar Şekerci, Emre Dünder

**Affiliations:** 1 Department of Psychiatric Nursing, Faculty of Health Sciences, Ordu University, Ordu, Türkiye; 2 Department of Mathematics and Statistics, College of Science, Imam Mohammad Ibn Saud Islamic University (IMSIU), Riyadh, Saudi Arabia; 3 Department of Public Relations, Faculty of Communication, Galatasaray University, Istanbul, Türkiye; 4 Department of Statistics, Faculty of Science, Ondokuz Mayis University, Samsun, Türkiye

**Keywords:** digital addiction, digital amnesia, mental health, public health, reliability, scale adaptation, validity

The Figure captions were in the wrong order in the PDF/HTML version of this paper. The captions for Figures 2, 3 were interchanged: the figure currently labeled “Figure 2” should bear the caption “Distribution of responses according to Digital Amnesia dimensions,” whereas the figure currently labeled “Figure 3” should bear the caption “Confirmatory factor analysis (CFA) diagram for the Turkish version of the Digital Amnesia Scale.” Consequently, the figure references in Section 2.5 (Data Analysis), Section 3.1 (Descriptive findings), and Section 3.3 (Construct validity) were incorrect. The order has now been corrected.

There was a mistake in the captions of Figures 2–4 and in the footnotes of Tables 1–3 as published. The abbreviation “digamn” was incorrectly defined in Turkish (“dijital amnezi”) instead of English. The corrected caption abbreviation appears below.

“digamn: digital amnesia”

There was a mistake in Table 2 as published. The subdimension was erroneously labeled “Digital dependency,” which is inconsistent with the subdimension name “Digital addiction” used throughout the rest of the manuscript (Sections 2.2.3, 3.2, Table 1, and figure footnotes). The corrected Table 2 appears below.

**Table 2:** Reliability analysis results of the Turkish version of the Digital Amnesia Scale.

Factor/item	CITC	CAIID	Alpha	Omega
Digital distraction				
digamn1	0.752	0.843	0.864	0.867
digamn2	0.457	0.871		
digamn3	0.703	0.849		
digamn4	0.728	0.846		
digamn5	0.787	0.838		
digamn6	0.669	0.852		
digamn7	0.685	0.851		
digamn8	0.798	0.837		
digamn9	0.637	0.857		
Digital addiction				
digamn10	0.613	0.695	0.726	0.749
digamn11	0.720	0.662		
digamn12	0.525	0.762		
digamn13	0.690	0.668		
digamn14	0.671	0.675		
digamn15	0.729	0.658		
Digital detox				
digamn16	0.675	0.659	0.713	0.718
digamn17	0.720	0.651		
digamn18	0.661	0.682		
digamn19	0.747	0.632		
digamn20	0.612	0.699		
Overall results			0.844	0.886

CITC, corrected item-total correlation; CAIID, Cronbach's alpha if item deleted; alpha, Cronbach's alpha; omega, McDonald's omega; digdist, digital distraction; digadd, digital addiction; digdet, digital detox; digamn, digital amnesia.

In the abstract, the reported range of unstandardized factor loadings was incorrect. The minimum value was erroneously reported as 0.742, whereas the correct minimum value

reported in Table 1 is 0.365 (item digamn12). The standardized factor loading range was also reported with insufficient precision. This has been corrected to read:

“Unstandardized factor loadings ranged between 0.365 and 1.371, while standardized factor loadings ranged between 0.211 and 0.845.”

A correction has been made to Section 3.3 (Construct validity), where the path coefficients were erroneously stated to be presented in Table 2; the path coefficients are reported in Table 1, not Table 2. The corrected sentence reads:

“The resulting path coefficients are presented in Table 1, and the model fit indices are presented in Table 3.”

A correction has been made to Section 2.2.2 (Digital Addiction Scale), where the Cronbach's alpha value reported for the Digital Addiction Scale in the present study (0.85) was inconsistent with the value reported in Section 3.2 (0.934). The corrected sentence reads:

“…and in the present study, it was found to be 0.934, indicating excellent internal consistency.”

A correction has been made to Section 2.3 of the Methods. The sentence describing the correlation strength classification thresholds was erroneously placed under Section 2.3.4 (Construct validity), where these criteria are not applicable. This sentence has been moved to the end of Section 2.3.5 (Criterion validity), where the criteria are used to interpret Pearson correlations.

The original version of this article has been updated.

